# Registration of Births, Deaths, and Marriages in Rhode Island for 1853

**Published:** 1854-11

**Authors:** 


*Registration of Births, Marriages and Deaths in Rhode Island, for the year ending May 31st,* 1853. Prepared under the direction of ASA POTTER, Secretary of State.

The above is a pamphlet of 188 pages, containing the first annual registration report for the State of Rhode Island.

The work of compiling the report was chiefly done by Thomas H. Webb, M. D., of Providence, who seems to have performed his task with much patience and care

Forty pages are occupied with statistical tables giving the number of Births, Marriages and Deaths in each county with their population, as determined by the census. These tables embrace a great amount of very useful matter. The remaining 148 pages are occupied with an interesting history of the progress of legislation, and a detailed analysis of the facts furnished in the statistical tables.

Dr. Webb has evidently bestowed on the report much labor, and has thereby made it correspondingly interesting and valuable to the whole profession. We hope the time will soon come when every State in the Union will have, not only a good registry law, but one well executed.

